# Supplementary material for: Functional and structural characterization of an ECF-type ABC transporter for vitamin B12
Source: eLife. 2018 May 29;7:e35828. doi: 10.7554/eLife.35828 (PMC5997447; doi:10.7554/eLife.35828)
Supplement: Supplementary file 1. [file elife-35828-supp1.docx]

**Supplementary table 1: Data collection, phasing and refinement statistics.**

|  | |
| --- | --- |
|  | **ECF-CbrT** |
|  | |
| **Data collection** |  |
| Space group | P 1 |
| Unit cell dimensions |  |
| *a, b, c* (Å) | 85.47, 92.86, 105.51 |
| *α, β, γ* (°) | 72.57, 66.27, 62.89 |
| Resolution range (Å) | 47.80, 3.40 |
| *R*_merge_ (%) | 17.7 (>100)* |
| *I*/*σI* | 3.82 (0.38)* |
| Completeness (%) | 93 (94) * |
| Redundancy | 3,68 (3,7)* |
|  |  |
| **Refinement** |  |
| Resolution (Å) | 3.4 |
| No. of reflections | 31753 |
| *R*_work_/*R*_free_ | 0.238/ 0.293 |
| Total no. of atoms | 15083 |
| R.m.s. deviations |  |
| Bond lengths (Å) | 0.010 |
| Bond angles (Å) | 1.487 |
|  | |

*Values in parentheses are for the highest-resolution shell.
