# Supplementary material for: Functional and structural characterization of an ECF-type ABC transporter for vitamin B12
Source: eLife. 2018 May 29;7:e35828. doi: 10.7554/eLife.35828 (PMC5997447; doi:10.7554/eLife.35828)
Supplement: Supplementary file 2. [file elife-35828-supp2.docx]

**Supplementary table 2:** **Primer list used in this study.**

|  | |
| --- | --- |
| Primer name | Sequence (5’ – 3’) |
|  | |
| CbrT_Ld_NcoI_frwd | TAACCATGGGACAGACCAAGGAACGCTAC |
| CbrT_Ld_cHis8_HindIII_rev | AATAAGCTTTCATTAATGATGATGGTGATGGTGGTGGTGAGCATTTTGCTTCCACCC |
| CbrT_Ld_XbaI_frwd | CCATCTAGATGCAGACCAAGGAACGCTACCAG |
| CbrT_Ld_XhoI_rev | AATCTCGAGTCATTAAGCATTTTGCTTCCACCCTGC |
| Seq_frwd | CTCTACTGTTTCTCCATACCCG |
| Seq_rev | GCTGAAAATCTTCTCTCATCCG |
|  | |
